# Genomic Surveillance of SARS-CoV-2 in Malaysia during the Era of Endemic COVID-19

**DOI:** 10.3390/life13081644

**Published:** 2023-07-28

**Authors:** Tze Yan Lee, Wai Feng Lim, Geik Yong Ang, Choo Yee Yu

**Affiliations:** 1 School of Liberal Arts, Science and Technology (PUScLST), Perdana University, Kuala Lumpur 50490, Malaysia; 2 Sunway Medical Centre, Bandar Sunway, Subang Jaya 47500, Malaysia; limwf@sunway.com.my; 3 Faculty of Sports Science and Recreation, Universiti Teknologi MARA, Shah Alam 40450, Malaysia; geikyong@uitm.edu.my; 4 Laboratory of Vaccine and Biomolecules, Institute of Bioscience, Universiti Putra Malaysia, Serdang 43400, Malaysia

**Keywords:** COVID-19, endemic, BA.5, XBB, subvariants, VOI, Malaysia, Omicron, waves, VUM

## Abstract

On 5 May 2023, WHO declared the end of coronavirus disease 2019 (COVID-19) as a public health emergency of international concern. However, the risk of new severe acute respiratory syndrome coronavirus 2 (SARS-CoV-2) variants causing rapid and high surges in cases and deaths remained. In Malaysia, five COVID-19 waves during the pandemic phase were well characterized, but similar studies focusing on the endemic phase were lacking. Hence, we retrieved 14,965 SARS-CoV-2 genomic sequences from the GISAID EpiCoV database for clade, lineage, and phylogenetic analysis in order to provide an insight into the population dynamics of SARS-CoV-2 that circulated in Malaysia from June 2022 to April 2023. The dominance of the Omicron variants was observed, and two new waves of infections driven by BA.5.2 and XBB.1, respectively, were detected. Data as of April 2023 also pointed to a possible eighth wave driven by XBB.1.9. Although new variants associated with higher transmissibility were behind the multiple surges, these subsequent waves had lower intensities as compared to the fourth and fifth waves. The on-going circulation and evolution of SARS-CoV-2 mean that COVID-19 still poses a serious threat, necessitating active genomic surveillance for early warning of potential new variants of concern.

## 1. Introduction

In late 2019, the emergent severe acute respiratory syndrome coronavirus 2 (SARS-CoV-2) was first detected in China [1], but the unprecedented rapid spread of the virus around the world led to the declaration of coronavirus disease 2019 (COVID-19) as a Public Health Emergency of International Concern (PHEIC) on 30 January 2020 [2] and a global pandemic on 11 March 2020 [3] by the World Health Organization (WHO). While severe acute respiratory syndrome (SARS) caused by SARS-CoV was contained in less than eight months from its onset in 2002, SARS-CoV-2 has continued to circulate widely as it enters the fourth year since its discovery [4]. On 5 May 2023, WHO declared that COVID-19 no longer constitutes a PHEIC given that the disease has become an established and ongoing health issue [5]. Globally, there has been a significant decline in the weekly number of COVID-19 related deaths, hospitalizations, and intensive care unit (ICU) admissions since the beginning of the pandemic, and these downward trends were attributed to the high population-level immunity to SARS-CoV-2 infection and/or vaccination, the consistent virulence of currently circulating Omicron sub-lineages, as compared to previously circulating Omicron sub-lineages and improved clinical case management [5].

As of 31 May 2023, 767 million confirmed COVID-19 cases have been reported globally, and more than 6.9 million deaths have been attributed to the disease [6]. The fact that there have been nearly 2 million new cases and over 12,000 deaths reported worldwide from 1 to 28 May 2023 [7] is a stark reminder of the continuing public health threat posed by COVID-19. Presently, the dominant circulating SARS-CoV-2 variant is the Omicron variant, as it accounts for more than 98% of sequences available since February 2022. The Omicron variant superseded the Delta variant following its emergence in late 2021 and was classified as a variant of concern (VOC) by WHO on 24 November 2021 [8,9]. In comparison to its predecessors (Alpha, Beta, Gamma, and Delta), Omicron, which belongs to the clade GRA, exhibits higher transmissibility and have the ability to evade the immune system, although it is less virulent [10]. There are currently five main lineages (BA.1, BA.2, BA.3, BA.4, and BA.5) and more than 700 sub-lineages of the Omicron variant [11]. However, the altered characteristics of some of these Omicron descendent lineages has prompted WHO to assign these sub-lineages independently as VOC, variant of interest (VOI), or variant under monitoring (VUM) [12]. As of 5 June 2023, XBB.1.5 and XBB.1.16 were classified as VOI whereas seven other Omicron sub-lineages (BA.2.75, CH.1.1, BQ.1, XBB*, XBB.1.9.1, XBB.1.9.2, and XBB.2.3) were classified as VUM [13].

In Malaysia, the Omicron variant was first detected in November 2021 and was implicated in driving the fifth COVID-19 wave that spanned from February to May 2022 [14]. The Omicron wave also resulted in the highest daily COVID-19 infection (33,496 cases), which surpassed the previous record (24,599 cases) during the Delta wave [15]. Nevertheless, the Omicron wave saw a significant reduction in the number of COVID-19-related hospital and ICU admissions, as well as in the number of COVID-19 patients requiring ventilator support [14]. Based on the successful implementation of the national COVID-19 vaccination program and the easing of pressure on the national healthcare system, the Ministry of Health Malaysia announced the transition of COVID-19 from pandemic to endemic phase on 1 April 2022 [16]. During the pandemic phase, the genetic epidemiology of SARS-CoV-2 in Malaysia has been investigated in detail and reported by multiple studies [14,17,18,19,20,21], but similar efforts have been found to be lacking since the country entered the endemic phase. Here, we report the characteristics of COVID-19 epidemiology and the phylodynamic of SARS-CoV-2 in Malaysia during the endemic period between June 2022 and April 2023 based on the genomic surveillance data that were deposited in the EpiCoV-Global Initiative on Sharing All Influenza Data (GISAID) database.

## 2. Materials and Methods

### 2.1. Epidemiological Datasets

The COVID-19 epidemiological data in Malaysia was retrieved from the KKMNOW portal [15] and its associated official GitHub content [22]. KKMNOW is an official portal developed by the Ministry of Health Malaysia and Department of Statistics Malaysia in support of making the data accessible to all, and the national COVID-19 database is updated on a weekly basis. All the information on the number of cases, deaths, and vaccinations up to 30 April 2023 was analyzed in this study.

### 2.2. Genome Analysis

The SARS-CoV-2 genomic sequences and associated metadata from Malaysia were retrieved from a publicly accessible EpiCoV database shared through the GISAID portal [23]. Sequences of samples collected between June 2022 and April 2023 with complete collection data and submitted by 1 May 2023 were retrieved and can be accessed at doi.org/10.55876/gis8.230527dw (accessed on 1 May 2023). All the sequences were further classified and analyzed based on the clade and lineage assignments.

### 2.3. Phylogenetic Analysis

For phylogenetic analysis, SARS-CoV-2 genomic sequences were downloaded from the EpiCoV database by applying the “complete” and “low coverage excluded” filtering functions. The EpiCov database defines “complete genome” as sequences with more than 29,000 nucleotides, whereas low coverage refers to sequences containing more than 5% ambiguous nucleotides (Ns). The phylogenetic tree was constructed using the Nextstrain SARS-CoV-2 workflow [24], and hCoV-19/Wuhan/WIV04/2019 (WIV04) was used as a reference sequence (GISAID EPI_ISL_402124). The result was then visualized on the Auspice server (https://auspice.us/; accessed on 10 May 2023).

## 3. Results and Discussion

**Figure 1:**
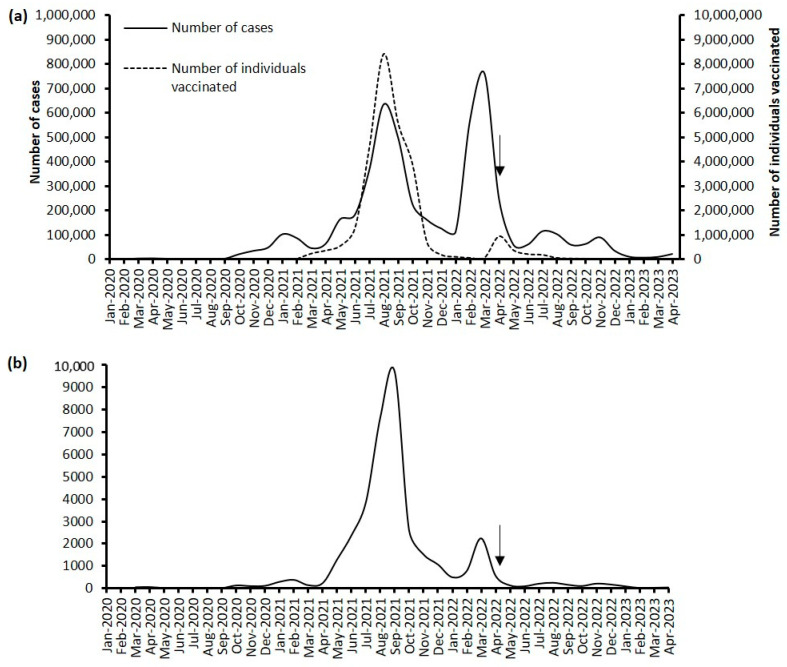
Epidemiology of COVID-19 in Malaysia as of 30 April 2023. (**a**) Number of confirmed cases and number of individuals fully vaccinated. (**b**) Number of deaths. The arrow indicates the transition from pandemic to endemic.

As of 30 April 2023, a total of 5,072,808 confirmed cases and 37,021 deaths had been recorded in Malaysia (Figure 1). Among the Malaysian population, which has an estimated size of 32.6 million [25], 27,504,570 individuals have been fully vaccinated (either a two-dose protocol or a single-dose protocol depending on the type of vaccine), and the number of fully vaccinated individuals accounted for 84% of the population. From June 2022 to April 2023, a total of 556,298 confirmed cases were reported, which represents 11.2% of all the cases that were detected in Malaysia as of April 2023. Two waves of COVID-19 (referred to as the sixth and seventh waves hereafter) were also identified during this period of endemicity, where the number of cases surged (June–September 2022 and October 2022–January 2023). The two waves recorded the highest number of cases in the months of July 2022 (113,988 cases) and November 2022 (89,204 cases), respectively, but these figures pale in comparison to the record high of 759,183 cases in March 2022 during the fifth wave [14]. Since February 2023, a surge in the number of confirmed cases has been observed, indicting the onset of a plausible eighth COVID-19 wave, with the number of monthly cases for April 2023 standing at 21,070.

**Figure 2:**
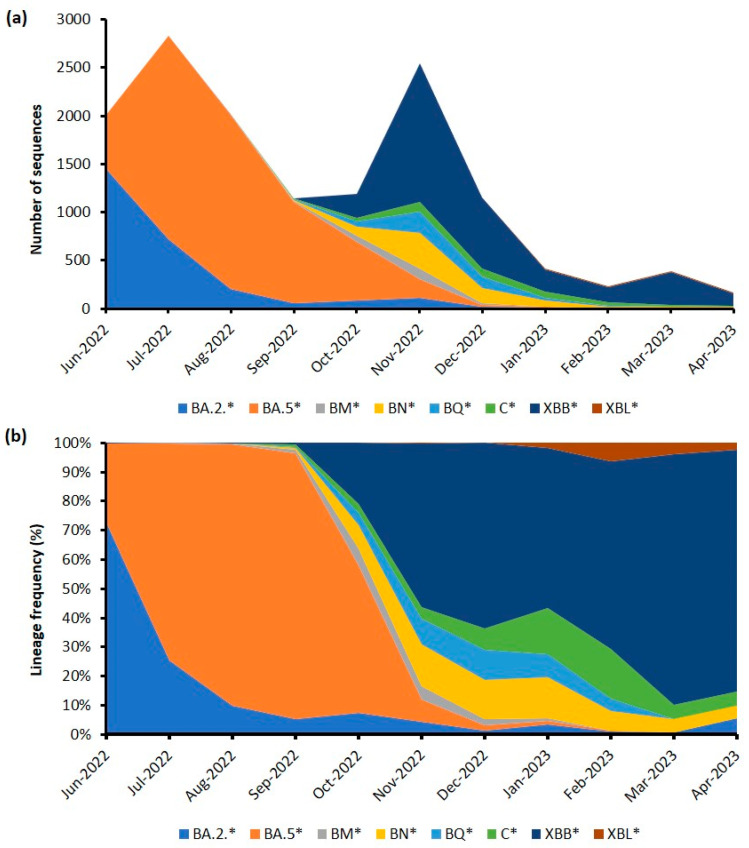
Chronological distribution of Pango lineages among the 14,965 SARS-CoV-2 genomic sequences in Malaysia. (**a**) Number of sequences based on Pango lineages. (**b**) Distribution of Pango lineage frequency. Only Pango lineages present in more than 5% of a month are shown. * Includes its sub-lineages.

A total of 14,965 SARS-CoV-2 genomic sequences from samples collected between June 2022 and April 2023 were retrieved from the EpiCoV database for analysis. The number of sequences retrieved represents 2.6% of the total number of confirmed cases reported during that period of time; a figure that is considerably higher than the previously reported percentage of SARS-CoV-2 genomic sequences submitted versus confirmed cases (0.42%) in Malaysia as of May 2022 [14]. The clade assignment of the genomic sequences retrieved showed that GRA (Omicron) continued to be the predominant clade and accounted for 99.7% of all the sequences submitted. A diverse set of lineages was detected, with up to 329 lineages identified from the retrieved SARS-CoV-2 genomic sequences. However, the majority of the sequences (82%) could be traced back to three lineages, whereas the frequency of all other lineages was less than 5% (Figure 2). The most prevalent lineage of samples collected between June 2022 and April 2023 was found to be BA.5 (42.4%), followed by XBB (21.8%) and BA.2 (17.8%).

**Figure 3:**
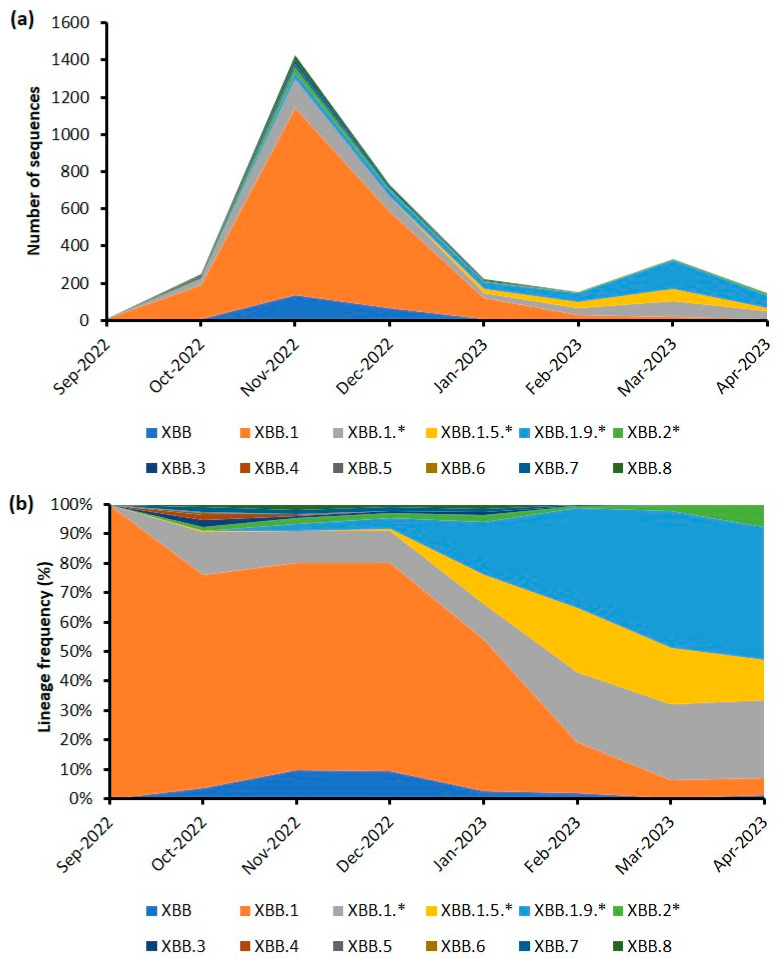
Chronological distribution of XBB lineages from September 2022 to April 2023 in Malaysia. (**a**) Number of sequences based on Pango lineages. (**b**) Distribution of Pangolineage frequency. Only lineages having more than five sequences are shown. * Includes their sub-lineages.

The emergence of BA.5 and its sub-lineages in February 2022 eventually led to the sixth wave as they displaced the BA.2 lineages that were predominant during the fifth wave. Even though there were 56 BA.5 sub-lineages that were detected during the endemic phase, BA.5.2 emerged as the predominant sub-lineage and accounted for 67.2% of all sequences belonging to BA.5 and its descendent lineages. A similar scenario was also reported in China, whereby BA.5.2 was found to be one of the main prevalent variants and subsequently became the predominant variant globally from July–October 2022 [11]. Other notable BA.5 sub-lineages detected during the endemic phase in Malaysia included BA.5.2.1 (9.9%), BA.5.2.28 (7.0%), BA.5.1 (3%), and BA.5.2.6 (2.4%). Following the sixth wave, the subsequent months saw further shifts in the dominant lineage. Between October 2022 and April 2023, dominance of XBB and its sub-lineages became apparent, with XBB.1 being the predominant sub-lineage (57.5%), followed by XBB.1.9.1 (7.2%), XBB (7.0%), and XBB 1.1 (7.0%). The sub-lineage distribution of XBB was further analyzed chronologically, as shown in Figure 3. The detailed analysis indicates that the seventh and plausible eight waves were associated with different XBB sub-lineages. The seventh wave was dominated by the XBB.1 sub-lineage, whereas the XBB.1.9 sub-lineage appeared to be driving the onset of the eighth wave.

Two currently circulating VOIs have also been detected in Malaysia. A total of 85 and 28 genomic sequences belonging to XBB.1.5 and XBB.1.16, respectively, were detected in Malaysia. The first case of XBB.1.5 was detected in October 2022, whereas XBB.1.16 was detected later, in March 2023. The XBB parent lineage is a result of a recombination event between two BA.2 lineages (BJ.1 and BM.1.1.1) [26] and is capable of posing a higher reinfection risk, transmissibility, and immune evasion [27,28]. In the United States of America, the XBB.1.5 has been found to spread rapidly, and the increase from 10% to 25% of the cases that the lineage is responsible for in a span of 4-weeks is said to be alarming [29,30]. XBB.1.5 has a characteristic S486P mutation in the spike protein, an event that is rare given that two substitutions of nucleotides in the same codon are required for the change in the amino acid to occur [31]. This amino acid change increases its binding affinity to the angiotensin-converting enzyme 2 receptor and thus enhances its infectivity [30]. Compared to XBB.1.5, XBB.1.16 contains two additional mutations in the spike proteins (E180V and K478R), which may confer a growth advantage [32]. A total of 3648 XBB.1.16 genomic sequences were reported in GISAID as of 17 April 2023, where India accounted for 63.4% of all the sequences [33]. In Malaysia, the recent introduction of the two VOIs might initiate another wave of infections, and this further highlights the importance of continuous SARS-CoV-2 genomic surveillance.

**Figure 4:**
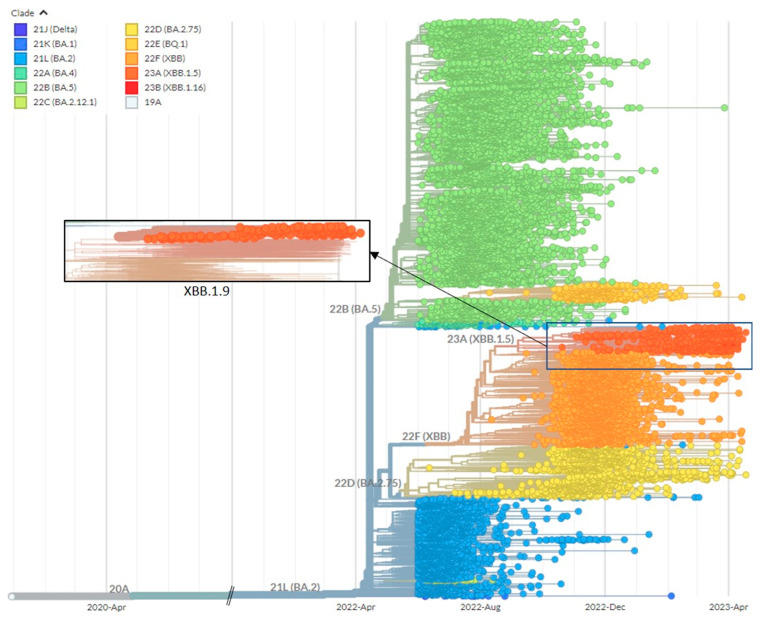
Phylogenetic tree constructed based on 12,370 SARS-CoV-2 genomic sequences in Malaysia spanning from June 2022 to April 2023. An inset showing only XBB.1.9 was provided.

Construction of the phylogenetic tree from 12,370 complete, high coverage genome sequences after filtering and removal of sequences by EpiCov (*n* = 2318) and Nextstrain SARS-CoV-2 workflow (*n* = 277) provides evidence of a progressive replacement of BA.2 by BA.5 (sixth wave), followed by XBB.1 (seventh wave), and XBB.1.9 (plausible eighth wave) (Figure 4).

## 4. Conclusions

In this study, we provide insights into the genomic epidemiology of SARS-CoV-2 in Malaysia during the endemic period spanning from June 2022 to April 2023. Two new COVID-19 waves were detected, albeit of lower intensity as compared to the fifth wave, and these were driven by BA.5.2 and XBB.1, respectively. The recent surge in the number of cases from February to April 2023 also points to the onset of the plausible eighth wave, and XBB.1.9 appeared to be the dominant driver behind this impending wave. This study served to highlight the importance of implementing continuous genomic surveillance efforts even after the pandemic is over, as the evolution of SARS-CoV-2 can lead to the emergence of new variants with altered pathogenicity, transmissibility, and immune evasiveness, causing new outbreaks worldwide.

## Data Availability

The findings of this study are based on metadata associated with 14,965 sequences available on GISAID up to 1 May 2023, and accessible at doi.org/10.55876/gis8.230527dw (accessed on 1 May 2023).
